# Teaching the Emergency Department Patient Experience: Needs Assessment from the CORD-EM Task Force

**DOI:** 10.5811/westjem.2016.9.30667

**Published:** 2016-11-08

**Authors:** Kory S. London, Jeffrey Druck, Matthew Silver, Douglas Finefrock

**Affiliations:** * Thomas Jefferson University, Sidney Kimmel Medical College, Department of Emergency Medicine, Philadelphia, Pennsylvania; † University of Colorado, Denver, Colorado; ‡ Kaiser Permanente, San Diego Medical Center, San Diego, California; § Hackensack University Medical Center, Hackensack, New Jersey

## Abstract

**Introduction:**

Since the creation of Hospital Consumer Assessment of Healthcare Providers and Systems (HCAHPS) patient satisfaction (PS) scores, patient experience (PE) has become a metric that can profoundly affect the fiscal balance of hospital systems, reputation of entire departments and welfare of individual physicians. While government and hospital mandates demonstrate the prominence of PE as a quality measure, no such mandate exists for its education. The objective of this study was to determine the education and evaluation landscape for PE in categorical emergency medicine (EM) residencies.

**Methods:**

This was a prospective survey analysis of the Council of Emergency Medicine Residency Directors (CORD) membership. Program directors (PDs), assistant PDs and core faculty who are part of the CORD listserv were sent an email link to a brief, anonymous electronic survey. Respondents were asked their position in the residency, the name of their department, and questions regarding the presence and types of PS evaluative data and PE education they provide.

**Results:**

We obtained 168 responses from 139 individual residencies, representing 72% of all categorical EM residencies. This survey found that only 27% of responding residencies provide PS data to their residents. Of those programs, 61% offer simulation scores, 39% provide third-party attending data on cases with resident participation, 37% provide third-party acquired data specifically about residents and 37% provide internally acquired quantitative data.

Only 35% of residencies reported having any organized PE curricula. Of the programs that provide an organized PE curriculum, most offer multiple modalities; 96% provide didactic lectures, 49% small group sessions, 47% simulation sessions and 27% specifically use standardized patient encounters in their simulation sessions.

**Conclusion:**

The majority of categorical EM residencies do not provide either PS data or any organized PE curriculum. Those that do use a heterogeneous set of data collection modalities and educational techniques. American Osteopathic Association and Accreditation Council for Graduate Medical Education residencies show no significant differences in their resident PS data provision or formal curricula. Further work is needed to improve education given the high stakes of PS scores in the emergency physician’s career.

## INTRODUCTION

In 1976, Ware, Snyder and Wright published the first rigorous and validated patient satisfaction (PS) healthcare questionnaire, the PSQ.^1^,^2^ Within a decade, two Notre Dame professors, Irwin Press and Rod Ganey, founded Press Ganey Associates whose mission of “improving the patient experience through compassionate, connected care” became the basis of a healthcare revolution.^3^ Hospitals saw the competitive advantage that could be gained by measuring their patients’ satisfaction and comparing these scores to other similar organizations. Service quality, as measured through PS scores, became a key component of measuring the quality and value of healthcare.^4^

As the single largest payer of healthcare dollars in the United States, the federal government followed suit. In 2002, through a partnership with the Agency for Healthcare Research and Quality (AHRQ), the Centers for Medicare and Medicaid (CMS) first developed and then implemented the Hospital Consumer Assessment of Healthcare Providers and Systems (HCAHPS) survey. As part of the Deficit Reduction Act of 2005, and further through the Patient Protection and Affordable Care Act of 2010, hospitals received financial incentives for participating in the HCAHPS survey. The HCAHPS data are not only used to provide financial incentives to hospitals, but are also publicly reported on the CMS’ consumer-oriented website,^5^ further emphasizing the import of these scores to hospital systems and their administrators.

Several studies have linked PS to improved outcome measures,^6^–^10^ but physicians are still skeptical of the link between satisfaction and quality. A well-publicized trial published by Fenton et al in 2012, further sparked the controversy, revealing that higher PS scores were associated with higher overall healthcare and prescription drug expenditures, and increased mortality.^10^

Despite the conflicting evidence, PS scores have become a key component in the metric-driven environment in which physicians practice today. The Accreditation Council for Graduate Medical Education (ACGME), through the Next Accreditation System and Milestones, developed a framework for the assessment of residents in each of several core competency areas.^11^ Included in the milestones are several competencies relating to how well residents connect with their patients, including professionalism, interpersonal and communication skills, and system-based practice. Residency programs will need to train their residents in effective communication strategies, educate them on the importance of PS scores and prepare them for a practice where metrics drive hospital reimbursement and physician performance assessment.

The objective of this study was to determine the education and evaluation landscape for patient experience in categorical emergency medicine (EM) residencies in the U.S.

## METHODS

The needs assessment survey was created using plain language and consensus questions developed by the authors and task force. In the interest of acquiring a large dataset, we kept the number of questions to a minimum to respect the varied duties of the respondents. Survey questions were tested for content and response process issues by the authors’ own departmental leadership prior to survey release. Further validity evidence was not collected. We collected data about participants’ departmental role and residency name, but that information was solely used to assist in culling duplicate program responses and to analyze ACGME vs. American Osteopathic Association (AOA) differences respectively. All data relating to identity were strictly separated from program responses. The institutional review board reviewed this study and deemed it exempt.

We obtained access to the faculty through the use of the Council of Residency Directors for Emergency Medicine (CORD-EM) faculty listserv. The CORD-EM membership includes the departments of categorical U.S. residencies, prospective U.S. residencies and select international EM residency programs. Specifically, the membership is restricted to program directors (PDs), assistant PDs and core faculty of the departments’ education divisions. While patient experience is an international movement, we decided to limit participation to categorical U.S. residencies that already exist.

The only inclusion criteria were that respondents had to work at currently running U.S. categorical residencies and participate in the CORD-EM faculty listserv. Exclusion criteria included international faculty and those of residencies not yet currently in operation. Given the likelihood of multiple responses from some institutions, it was decided that in the case of heterogeneity, the most senior respondent’s data would be used (PD>APD>core faculty).

The listserv contains 194 residencies that split into 30 AOA or joint AOA/ACGME accredited programs and 164 ACGME accredited programs. The AOA and joint accredited programs were combined for analysis given AOA accreditation was the variable being studied. The surveying itself was performed using the online survey service SurveyMonkey®. An initial attempt at data collection was made by a form email sent through the listserv. When responses began to decrease, we sent a second form email through the listserv to encourage those who had overlooked the first request. Finally, individual program directors from non-responsive departments were sent targeted emails asking for participation during the third and final round of data collection.

The authors analyzed data using the built-in tools from SurveyMonkey and Microsoft Excel. We performed comparison between AOA and ACGME programs using chi-square testing with p values set a 0.05.

## RESULTS

We received a total of 168 individual responses from 139 programs. This represents a program participation rate of 72%. Of the 139 programs that provided data, 15 were AOA accredited, 119 were ACGME and five were joint AOA/ACGME. This represents 62.5% of AOA residencies that participate in CORD-EM, 72% of ACGME residencies and 83% of joint accreditation programs. There was no significant difference in rates of response between AOA/joint and ACGME programs (p=0.51).

Of those 168 responses, 107 were by PDs, 46 by APDs and 15 by academic core faculty. Given multiple responses by 29 programs, the final participant count was 107 PDs (77%), 24 APDs (17%) and eight academic core faculty (6%). No program had >2 responses. Categorical EM programs exist in 43 states and Puerto Rico. We obtained responses from 41 of those.

This survey found that only 27% of responding residencies provide any PS data to their residents. Of those programs, most offer multiple modalities; 37% provide internally acquired quantitative data, 21% provide internally acquired anecdotal data, 37% provide third-party metrics specifically about residents, 39% provide third-party attending metrics about resident cases, 61% provide simulation scores (quantitative data taken from simulation encounters), and 21% use other modalities.

Only 35% of residencies provided any organized patient experience (PE) curriculum. Of these programs, again, most offer multiple modalities: 96% provide didactic lectures, 49% small group sessions, 47% simulation sessions. and 27% specifically use standardized patient encounters in their simulation sessions. Finally, 35% provide online or asynchronous resources for their residents. There was no significant difference in numbers of AOA and ACGME programs providing curicula (p=0.32).

Of the programs that do provide PE education, 47% describe the differences between different PS surveys. Again, there was no significant difference between AOA and ACGME programs (p=0.27). Finally, 100% of all programs who provide PE curiculum describe methods to improve PS scores.

## DISCUSSION

Our study demonstrates that residency programs do not have a uniform approach to resident instruction on PE training or satisfaction measurement, with 65% of all residency programs having no formal curriculum on PE at all. Other aspects of communication have also been assessed in resident education and seem to occur more consistently than those focused on the patient experience. In a recent study by Hern et al, 57% of residency programs had curriculum focused on transitions of care. Hern et al recently found 57% of residency programs have curriculum focused on handoffs, a much higher percentage than PE.^12^ Another study found 93% of residency programs had curriculum focused on operations and administration. 13

AOA and ACGME rates were similar and suboptimal. There were insignificant trends showing AOA as better at providing scores/educating their residents. This will likely only fall farther down the list of AOA program priorities given the preparation required for their merger with the ACGME, due in 2020.

Why is PE training a neglected area of medical education? Although a relatively new topic in medical care, private practice emphasis and incentive-based compensation have skewed dramatically towards focusing on PS scores.^14^ It is possible that as academic institutions have been slower to emphasize this, it has taken longer to introduce this critical element to residency education. Only 37% of programs provided resident-specific survey information about PE data; in private practice, almost all facilities provide provider-specific patient data in the form of PS scores. It is also possible that academic practitioners may discount the value of patient satisfaction, as there is controversy as to the usefulness of PS scores as a corollary for excellent care. Alternatively, as PE is a relatively new field, there is less definitive evidence regarding the elements that contribute to a successful patient experience, possibly making educators less willing to teach on a subject they know little about and believe has been inadequately studied.

## LIMITATIONS

Our study does have a number of limitations. First, our response rate was not universal. Most likely, the bias associated with this response rate would be towards responders being more likely to have curriculum, and as a result, we expect that our results overestimate the implementation of curriculum and data collection for residents. In addition, we had 29 instances where two faculty members of the same residency program responded. Of the 29 programs, 13 had concordant responses (45%) and another four had the same responses except with respect to a single question (14%). This leaves 12 others with large and varied degrees of disagreement (41%). This variance has a minimal effect on the overall statistics, but it does deserve further evaluation. While the ultimate cause for this discordance is unclear, this likely represents evidence of a paucity of focus on PE in EM GME.

## CONCLUSION

The overall message of our study is the need for a more robust emphasis on patient experience education for EM residents. As PS is an element that physicians are being judged upon and penalized for, EM residencies are doing their residents a disservice by not preparing them adequately for clinical practice. We hope future research on PS will demonstrate best practices in resident education and further national standardization on curricular elements that help to improve the EM patient experience and EM physician patient-satisfaction scores.
